# High Prevalence of Iron Deficiency Exhibited in Internationally Competitive, Non-Professional Female Endurance Athletes—A Case Study

**DOI:** 10.3390/ijerph192416606

**Published:** 2022-12-10

**Authors:** Stacy T. Sims, Kelsi Mackay, Alana Leabeater, Anthea Clarke, Katherine Schofield, Matthew Driller

**Affiliations:** 1 WHISPA High Performance Sport New Zealand, Auckland 0632, New Zealand; 2 Te Huataki Waiora, Faculty of Health, Sport and Human Performance, University of Waikato, Hamilton 3216, New Zealand; 3 Sport, Performance, and Nutrition Research Group, School of Allied Health, Human Services and Sport, La Trobe University, Melbourne 3086, Australia

**Keywords:** REDs, energy deficiency, anaemia, women’s health

## Abstract

Background: While iron deficiency is commonly discussed in populations of professional female athletes, less is known about highly trained, sub-elite female athletes (e.g., those winning international age-group competitions) who generally have less access to medical and allied health support. Methods: Thirteen non-professional highly trained female endurance athletes provided training diaries and completed a blood test, where iron markers of haemoglobin (Hb), haematocrit (Hct), C-reactive protein (Crp), serum iron, serum ferritin, and transferrin were assessed. Resting metabolic rate (RMR) and body composition using dual-energy X-ray absorptiometry (DXA) were also obtained. Participants were classified as iron deficient (ID) if serum ferritin was <30 ug/L serum ferritin. Results: Six of the 13 females were classified as ID. Serum iron, ferritin, Hb, Hct, and ferrin were greater in the ID group (*p* < 0.05). Crp resulted in *large* to *very large* correlations with serum iron (*r* = −0.72), serum ferritin (*r* = −0.66), and transferrin (*r* = 0.70). Conclusions: In this population of highly trained female athletes, 46% were diagnosed with sub-optimal iron levels, which could have lasting health effects and impair athletic performance. The need for more education and support in non-professional athletes regarding iron deficiency is strongly advised.

## 1. Introduction

Iron deficiency is a common health issue in endurance sports, and it is reported to affect up to 3–11% of male athletes and 15–35% of female athletes [1,2,3]. Iron loss is exaggerated in athletes due to high training loads and the resulting haemolysis, sweating, and gastrointestinal bleeding, which cause the breakdown of red blood cells and the loss of iron [4]. Female athletes also lose blood during menstruation and, as a result, typically have higher dietary iron needs [5]. In addition to this, hepcidin, an iron-regulating hormone, is upregulated following exercise as part of the acute inflammatory response, which impairs the absorption of iron [6]. Given the importance of power-to-weight-ratio and the perceived benefit of low body mass in endurance sports, the incidence of low energy availability is also somewhat common amongst endurance athletes [7]. While low energy availability can have long-term effects on bone mineral density and general health, having a generally low energy intake in relation to the needs of the individual likely also increases the chance of having insufficient dietary iron. Given the known influence of low iron stores on an athlete’s capacity to train, perform, and recover [8], monitoring and managing iron deficiency is important in athletic populations. 

It is particularly important that, not only are athletes consuming sufficient iron in their diet, but that the timing of this intake is also considered around their training schedule [9,10]. Often, this requires careful consultation with a sports dietitian to ensure that appropriate iron levels can be maintained throughout the training and competition cycle by improving dietary intake of iron or introducing iron supplements, if appropriate [4]. The access to sport dietitians afforded to high-performance athletes allows for thorough education, diagnosis, and treatment of conditions, such as iron deficiency (with or without anaemia). Professional medical support is pertinent in the management of this condition, as incorrect iron supplementation can lead to gastrointestinal discomfort or toxic levels of iron at the liver [4]. 

While previous research has established the prevalence of iron deficiency in populations of elite female endurance athletes [11], less is known about non-professional, sub-elite female athletes, who often maintain a comparable training volume and performance level to elite athletes. For example, sub-elite and elite female runners complete a similar amount of training sessions and distance per week, but may exhibit differences in training intensity [12]. However, sub-elite athletes are less likely to receive dietary counselling than their elite counterparts [13], which may contribute to a poor understanding of the signs and symptoms of iron deficiency and the subsequent health and performance implications [14,15,16]. Therefore, the aim of the current study was to investigate the prevalence of iron deficiency in a non-professional, highly trained population of female endurance athletes, and to compare iron status to physical characteristics (e.g., body composition), training, blood markers, and other energy intake and expenditure-related variables. It is this population that is of particular interest given the limited support systems in place, despite a generally high volume of training and the potential risk of low energy availability. 

## 2. Materials and Methods

### 2.1. Participants 

Thirteen non-professional, highly trained, premenopausal female endurance athletes (mean ± SD: age: 32 ± 7 y) completed this study. Inclusion criteria included: (a) regular menstrual cycles (24–35 days in length) or using a contraceptive (monophasic pill or intrauterine device) for >6 months before entering the study, (b) representing their country at an international level within the past three years and currently participating on a regular basis in competitive endurance-sport activity (running, cycling, triathlon), (c) having no current or recent (<3 months) injury or illness, or chronic disease, (d) not being pregnant, (e) not smoking, and (f) not being previously diagnosed as iron deficient or currently taking iron supplements. All participants provided informed written consent, and the study received ethical approval from the Institutions Human Research Ethics Committee. 

### 2.2. Training Diaries 

Participants’ training was recorded over a seven-day period. Online platforms were used to track exercise (TrainingPeaks, Boulder, CO, USA). Total training duration and duration at high intensity (above 80% HR_max)_ were calculated upon analysis of training logs using heart rate-based training impulse (TRIMP). 

### 2.3. Blood Sampling

Blood samples were collected following an overnight fast (6:30 p.m. to 8:00 a.m.). All blood samples were obtained via the antecubital vein while in a seated position. After drawing blood, serum and plasma samples were obtained after centrifugation (3000 rpm, 10 min, 4 °C) and stored at −80 °C until analysed. Cortisol, iron-status variables (haemaglobin, haematocrit, iron, ferritin, transferrin), and a marker of inflammation (Crp) were analysed at the local health-board medical laboratory. Cortisol concentrations were measured by a sensitive and specific liquid chromatography-tandem mass spectrometry assay, with minor modifications to improve assay specificity. Athletes were classified as iron deficient (ID) if they had a ferritin <30 ug/L. We selected this threshold based on previous recommendations of other authors and clinicians [17,18,19].

### 2.4. Resting Metabolic Rate (RMR)

Measured RMR was conducted using the ‘RMR’ function on the TrueOne metabolic analyser (TrueOne 2400, Parvo Medics, Sandy, UT, USA). On the morning of the assessment, participants arrived at the laboratory for their scheduled lab appointment (between 0600 and 0830 h) following an overnight fast of >10 h, with participants refraining from exercise, caffeine, and alcohol intake <24 h prior to testing. Participants were instructed to sleep 8 h the night before testing. Participants arrived at the laboratory in a relaxed and rested state and were instructed to perform minimal activity between waking and arrival to the RMR testing session. Upon arrival to the laboratory, participants’ height (cm) and body mass (kg; in minimal clothing) were measured before a 5 min passive rest period prior to the RMR assessment. Participants were asked to relax and lie supine on a bed with the head rest set at an incline of 45° during the 20 min assessment period, but to maintain alertness with eyes open. The canopy was then placed over the head, shoulders, and upper chest of the participant to reduce contaminant air entering or expired air escaping during measurement. Flow rate was established at 28 to 30 mL.min^−1^ within the first 4 min of the assessment, as per the manufacturer’s instructions. Expired air was sampled every 15 s and averaged over 30 s. Data files were exported in Microsoft Excel format for subsequent calculation of RMR (kcal·day^−1^) via the Weir formula [20]. RMR was calculated using the average of the final minute of each 5-min segment of the assessment (4 to 5, 9 to 10, 14 to 15 and 19 to 20 min). The metabolic analyser and RMR protocol implemented in the current study has previously been shown to be reliable in a similar cohort of athletes [21]. 

### 2.5. Body Composition Analysis

Body composition and bone health measures were evaluated using whole-body dual-energy X-ray absorptiometry (DXA) scans (GE Prodigy Advance, GE Healthcare, Madison, WI, USA), at a private clinic. A total-body scan, in standard scan mode using the software enCORE (version 17), was performed and analysed by the same trained technician using standardized protocols. Body composition variables selected included percentage body fat % and bone mineral density (BMD, g/cm^2^) at the anterior-posterior lumbar spine (L1–L4) and total hip (mean result of two femur measurements). 

### 2.6. Statistical Analysis 

All data are presented as mean ± SD unless stated otherwise. Statistical significance was set at *p* < 0.05. Comparison of the ID and non-ID groups was achieved using a Student’s unpaired t-test and Cohen’s effect sizes (*d*). The magnitude of effect sizes were assessed with the following thresholds: <0.2, *trivial*; 0.2–0.6, *small*; 0.6–1.2, *moderate*; 1.2–2.0, *large*; 2.0–4.0, *very large*; >4.0, *extremely large* [22]. Pearson’s correlations (*r*) were used to examine associations between variables. The magnitude of correlation between variables was assessed with the following thresholds: <0.10, *trivial*; 0.10–0.30, *small*; 0.31–0.50, *moderate*; 0.51–0.70, *large*; 0.71–0.90, *very large*; and 0.91–1.0, *almost perfect* [23].

## 3. Results

Of the 13 women who were included in this study, six were classified as ID, and seven were classified as non-ID [24]. No individual was classified as being anaemic (Hb < 12 g·L^−1^). Participant anthropometric and training characteristics were similar between groups (Table 1). Table 2 presents the haematological profiles, RMR, and BMD of both groups. Those in the ID group reported elevated Crp (*p* = 0.0001, *d* = −3.33 ± 0.93, *very large*) and transferrin (*p* = 0.004, *d* = −1.82 ± 0.91, *large*). Serum ferritin (*p* = 0.002, *d* = 1.97 ± 0.92, *large*), serum iron (*p* = 0.011, *d* = 1.55 ± 0.91, *large*), haemoglobin (Hb) (*p* = 0.036, *d* = 1.24 ± 0.94, *large*) and haematocrit (Hct) (*p* = 0.034, *d* = 1.27 ± 0.92, *large*) were all significantly lower in the ID group when compared with the non-ID group. 

Correlations between variables are shown in Table 3. Crp was positively correlated with cortisol (*r* = 0.31) and transferrin (*r* = 0.70), as well as negatively correlated with serum ferritin (*r* = −0.66), iron (*r =* −0.72), and RMR (*r* = −0.52). Total training duration and high intensity training had *moderate* positive correlations with cortisol (*r* = 0.31–0.48) and *moderate* negative correlations with RMR (*r* = −0.66–−0.34). BMD was also associated with a *moderate* positive correlation with high intensity training duration (*r* = 0.68). 

## 4. Discussion

The current study provides an insight into the health profiles of a group of non-professional, highly trained female endurance athletes. Almost half of this group was identified as being iron deficient (<30 ug/L serum ferritin), despite having no differences in weekly training hours, or RMR between those classified as ID or not. The Crp and transferrin of those with ID were greater than those in the non-ID group. Both groups reported Crp values above what would be considered normal in a clinical setting, while only the ID group had a higher than normal transferrin value. Crp was also found to correlate with a number of iron profile variables, as well as cortisol. It is important to understand the influence of high-level training and performance on athletes and how this may present in haematological testing. While within clinical ‘normal’ ranges, the low iron status observed in the ID group would likely impact their training and performance [8,25], and individuals’ training status should be taken into consideration by general medical practitioners to ensure appropriate identification of iron deficiency in non-professional, highly trained female athletes. 

The prevalence of ID in this population is similar to previously reported incidences in other populations. Comparing those who were classified as ID vs. non-ID, there were no differences in training load volume, anthropometric characteristics, or iron intake. Both groups reported an iron intake below the recommendations for females (18 mg/day) [5], but were similar to other female athlete groups [26,27]. Potentially then, reasons that an individual may become iron deficient within this group of international level, non-professional athletes may be more specific to the type and timing of iron sources within the diet. Given the complex relationships and interactions between dietary sources of iron, dietary inhibitors, enhancers of iron absorption, and exercise-related hepcidin release, consultation with a dietitian is recommended to navigate this area. 

While this study uses the athlete-focussed definition of ID as being <30 ug/L, if assessed using clinical reference ranges, none of the athletes in this study would be identified as ID. However, within athletic populations a reference range of 30–99 ug/L has been classified as ‘functional iron deficiency’, where performance (but not health) may still be affected [28]. Indeed, while the non-ID group did not reach our threshold of ID in this study, the mean ferritin of 40 ug/L is still on the low end and worth continual monitoring. Given these non-professional athletes do not usually have their own medical team, as would be provided to professional athletes, they are required to go through the normal healthcare system. As such, these athletes could remain undiagnosed in normal healthcare settings based on the clinical guidelines for ID. However, given that the early stages of ID could have a disadvantageous effect on aerobic exercise performance [8], routine monitoring of iron levels in athletes with reference to athlete-specific ID definitions is recommended. Indeed, Sim et al. [29] recommended quarterly screening for iron deficiency in endurance athletes, with or without evidence of low energy availability, fatigue, or performance impairment.

Both groups in this study had Crp levels above the clinical reference range. However, the ID group had a mean Crp three and a half times higher than the non-ID group. Long term participation in exercise is known to have an anti-inflammatory response, which lowers Crp. Furthermore, a short-term inflammatory response does occur following exercise [30]. This short-term elevation can occur up to 48 h post exercise, so while this study controlled for exercise to not occur within 24 h of blood collection, it is possible that Crp was still elevated due to previous training. There is no known influence of ID on Crp response, so reasons for the differences between groups is currently unknown. Given ferritin is typically elevated during inflammation, higher Crp values may also increase the risk of iron deficiency being undiagnosed due to these transient changes. 

Endurance athletes experiencing high training loads often present with elevations in the stress hormone cortisol, indicating physiological disturbance at the hypothalamic-pituitary level [31,32]. Similarly, reductions in RMR may be observed after periods of intensified training and can be used as a diagnostic marker of low energy availability in athletes [33,34]. In the present study, training load (hours and intensity) was positively correlated with cortisol and negatively correlated with RMR, suggestive of training-related distress across the sample of athletes, regardless of iron status. This may be related to a poor balance between ‘intense’ training (>80% HR max) and low-intensity training in these athletes and a resultant increase in physiological strain [35]. Therefore, holistic monitoring of factors, such as training load, RMR, and stress hormones, in addition to iron status, may be worthwhile in populations of sub-elite female athletes. 

The small sample size in the current study is a limitation and does not allow for generalized inferences to a wider population of sub-elite female athletes; rather, it is designed to provide an insight into the possible prevalence of iron deficiency in this non-professional athletic population. A further limitation of the current study was the lack of dietary analysis. We would recommend that, in future studies, a full diet diary and dietary analysis is completed to identify any differences between ID and non-ID groups.

## 5. Conclusions

This research has identified a high prevalence of iron deficiency in sub-elite female athletes and adds to the evident need for an athlete-centred set of guidelines when identifying health issues such as iron deficiency. To avoid long-term health problems and enhance athletic performance, sub-elite athletes that are training and competing at high levels may benefit from targeted education around the benefits of regular monitoring of iron status and maintaining an adequate energy balance. There is also a need to educate both athletes and medical professionals of athlete-specific requirements in relation to monitoring iron stores. 

## Figures and Tables

**Table 1:** Participant characteristics ((grouped by iron status, iron deficient (ID) and non-iron deficient (non-ID)) *.

	ID (n = 6)	Non ID (n = 7)
Age (yrs)	31 ± 3	34 ± 10
Height (cm)	170.7 ± 2.4	164.2 ± 6.8
Body Mass (kg)	62.6 ± 7.5	58.8 ± 5.7
Body Fat (%)	21.0 ± 6.2	18.8 ± 5.0
BMI	21.6 ± 2.3	21.9 ± 1.7
Training (hrs/wk)	17 ± 5	20 ± 3

* Note: Iron deficiency (ID) defined as <30 ug/L serum ferritin. BMI—body mass index. Data shown as mean ± SD.

**Table 2:** Comparison of haematological profiles, bone mineral density (BMD), and resting metabolic rate (RMR) in female iron deficient (ID) athletes and non-iron deficient (non-ID) athletes.

Marker	Normal Ranges ^	ID (Mean ± SD)	Non ID (Mean ± SD)	*p*-Value (*p*)	Effect Size (Mean ± 90% CI)
Serum Ferritin (ug/L)	15–200	27.5 ± 2.8	41.0 ± 7.3	0.002 *	1.97 ±0.92 *(large)*
Serum Iron (mmol/L)	11–29	20.5 ± 1.0	25.5 ± 3.4	0.011 *	1.55 ±0.91 *(large)*
Transferrin (g/L)	2.1–3.6	3.9 ± 0.1	3.1 ± 0.6	0.004 *	−1.82 ±0.91 *(large)*
Crp (mg/dL)	<0.5	2.8 ± 0.5	0.8 ± 0.6	0.0001 *	−3.33 ±0.93 *(very large)*
Cortisol (nmol/L)	251–552	359.3 ± 112.4	320.8 ± 96.4	0.373	−0.47 ±0.95 *(unclear)*
Hb (g/L)	120–160	130.5 ± 7.8	143.8 ± 8.4	0.036 *	1.24 ±0.94 *(large)*
Hct (g/L)	0.355–0.449	0.392 ± 0.01	0.430 ± 0.02	0.034 *	1.27 ± 0.92 *(large)*
BMD (g/cm^2^)		1.2 ± 0.1	1.2 ± 0.1	0.70	−0.20 ±0.96 *(unclear)*
RMR (kcal)		1581.7 ± 84.2	1670.9 ± 247.9	0.42	0.43 ±0.91 *(unclear)*

* Indicates statistical significance (*p* < 0.05). ^ Normal ranges, as provided by the clinical laboratory where testing took place.

**Table 3:** Pearson’s correlations between measured blood markers, resting metabolic rate (RMR), and bone mineral density (BMD) in highly trained, non-professional female athletes.

	Body Mass	% BF	RMR	Cortisol	Serum Ferritin	Serum Iron	Transferrin	Crp	BMD	Age	Hct	Hb	Training hrs/wk
% BF	0.60 **												
RMR	0.14	−0.09											
Cortisol	0.41 *	−0.01	−0.49 *										
Serum Ferritin	−0.21	−0.32 *	0.11	0.06									
Serum Iron	0.03	−0.21	0.45 *	−0.11	0.70 **								
Transferrin	0.40 *	0.38 *	−0.48 *	0.53 **	−0.23	−0.45 *							
Crp	0.04	0.13	−0.52 **	0.31 *	−0.66 **	−0.72 ***	0.70 **						
BMD	0.66 **	0.23	0.12	0.48 *	0.03	0.16	0.16	−0.11					
Age	0.02	0.18	−0.20	−0.13	0.37 *	0.31 *	0	−0.3	−0.37 *				
Hct	−0.24	−0.21	0.08	−0.15	0.81 ***	0.71 ***	−0.21	−0.44 *	−0.19	0.32 *			
Hb	−0.09	−0.13	0.15	−0.22	0.75 ***	0.74 ***	−0.22	−0.5	−0.19	0.45 *	0.95 ***		
Total training duration	0.10	−0.23	−0.66 **	0.31 *	0.13	0.04	0.2	0.25	0.06	0.26	0.27	0.27	
High-intensity duration	0.30 *	−0.13	−0.34 *	0.48 *	0.18	0.24	0.15	0.03	0.68 **	−0.06	0.1	0.12	0.70 **

Magnitude of correlation = * *moderate*, ** *large*, *** *very large*. BF—body fat, RMR—resting metabolic rate, Crp—C-reactive protein, Hb—haemoglobin, Hct—haematocrit, BMD—bone mineral density.

## Data Availability

The data presented in this study are available on request from the corresponding author.
